# Changes in brain Glx in depressed bipolar patients treated with lamotrigine: A proton MRS study

**DOI:** 10.1016/j.jad.2018.12.092

**Published:** 2019-03-01

**Authors:** Beata R. Godlewska, Uzay E. Emir, Charles Masaki, Theodoras Bargiotas, Philip J Cowen

**Affiliations:** a Psychopharmacology Research Unit (PPRU), University Department of Psychiatry, University of Oxford, Neurosciences Building, Warneford Hospital, Oxford OX3 7JX, United Kingdom; b Oxford Centre for Functional MRI of the Brain, Nuffield Department of Clinical Neurosciences, University of Oxford, John Radcliffe Hospital, Oxford OX3 9DU, United Kingdom; c Oxford Health NHS Foundation Trust, Warneford Hospital, Warneford Lane, Oxford OX3 7JX, United Kingdom

**Keywords:** Bipolar disorder, Bipolar depression, Lamotrigine, MRS, Glutamate, Glx

## Abstract

**Background:**

Lamotrigine is a useful treatment in bipolar depression but requires several weeks of dose titration before its clinical effects can be assessed. Animal experimental studies suggest that lamotrigine lowers glutamate release. The aim of the current study was to assess the effect of lamotrigine on brain glutamate in depressed bipolar patients and to determine whether baseline glutamate could be used to predict clinical response.

**Methods:**

We studied 21 bipolar patients who received lamotrigine treatment for a current episode of depression. Before starting lamotrigine and after 10–12 weeks treatment, patients underwent proton magnetic resonance spectroscopy (MRS) scanning at 3 Tesla where levels of glutamate (measured as Glx) were determined in anterior cingulate cortex (ACC).

**Results:**

Overall, lamotrigine treatment had no significant effect on Glx levels in ACC. However, in patients who responded clinically to lamotrigine treatment Glx concentrations were significantly increased. Baseline levels of Glx did not predict response to lamotrigine.

**Limitations:**

The main limitation of the study was the modest sample size. Most patients were medicated which may have modified the effect of lamotrigine on glutamate activity. MRS at 3T cannot give a reliable estimate of glutamate separate from its main metabolite, glutamine, and thus changes in Glx may not give a precise estimate of effects of lamotrigine on glutamate itself.

**Conclusion:**

Lamotrigine does not appear to have a direct effect on glutamate levels in ACC in bipolar patients. However, therapeutic improvement during lamotrigine was associated with increased Glx, suggesting that alterations in glutamatergic activity might be related to recovery from bipolar depression.

## Highlights

• MRS was used to measure determine brain glutamate (measured as Glx) in depressed bipolar patients before and after 12 weeks of lamotrigine.
• Overall, lamotrigine treatment had no significant effect on Glx levels in anterior cingulate cortex (ACC).
• However, clinical improvement with lamotrigine was associated with an increase in Glx.
• Baseline levels of Glx did not predict response to lamotrigine.

## 1 Introduction

Recurrent depression is the one of the main burdens experienced by patients with bipolar disorder. The treatment of bipolar depression is often difficult, with conventional antidepressants generally not recommended, being relatively ineffective and also carrying the risk of mood destabilisation (Bowden and Singh, 2016). The anticonvulsant drug, lamotrigine, has evidence of efficacy in both acute bipolar depression and in the longer-term prevention of depression in bipolar disorder (Solmi et al., 2016). However, as with other pharmacological treatments in bipolar disorder, lamotrigine is effective in only a proportion of patients. Moreover, the risk of Stevens-Johnson syndrome associated with lamotrigine treatment mandates a particularly slow initiation of dosing (Wang et al., 2015). Hence assessment of clinical response may take many weeks.

There would therefore be significant utility in the identification of a biomarker that could predict therapeutic response to lamotrigine treatment in bipolar patients. Development of such a biomarker could be based on knowledge of the mode of action of lamotrigine to alleviate depression in bipolar illness. Lamotrigine's pharmacological actions are complex and effects on ion channels and various neurotransmitters have been reported. However, an action of potential relevance in bipolar depression is reduction in glutamate release probably secondary to inhibition of voltage-sensitive sodium channels leading to stabilization of presynaptic neuronal membranes (Ketter et al., 2003).

Glutamate is the main excitatory neurotransmitter in the brain, whose removal from the synapse depends on the integrity of glial cells. A number of lines of evidence have implicated glutamate in the pathophysiology of mood disorders. For example, in addition to lamotrigine, a number of drugs acting on glutamate mechanisms have antidepressant effects, most notably the NMDA receptor antagonist, ketamine. Currently employed mood stabilising agents such as lithium and valproate modify glutamate release and glutamate receptor function. Further, post-mortem studies of mood disorders patients have reported loss of glial cells together with changes in expression of glutamate receptors (see Sanacora et al., 2008, Yuksel and Ongur, 2010).

Glutamate levels in the human brain can be measured non-invasively by proton magnetic resonance spectroscopy (MRS) (Yuksel and Ongur, 2010). While the findings are somewhat inconsistent a meta-analysis suggested that glutamate concentrations are increased in frontal brain regions, in bipolar illness (Gigante et al., 2012). In this study we therefore predicted that lamotrigine treatment would lower MRS glutamate levels in bipolar patients. We also predicted that higher baseline levels of glutamate would be associated with a better clinical outcome of lamotrigine treatment because high baseline glutamate might identify a specific subgroup of patients who would be helped by the glutamate-lowering effect of lamotrigine.

## 2 Experimental procedures

### 2.1 Study setting

Participants with bipolar disorder were patients of the Oxford Health NHS Foundation Trust, identified by their treating clinicians as being depressed and for whom treatment with lamotrigine was clinically indicated. Patients about to start lamotrigine were approached by a member of the research team and asked if they would give consent to an MRS examination before and during the course of lamotrigine treatment. All participants gave full, written informed consent to the study which was approved by the Hampshire A Research Ethics Committee.

### 2.2 Participants and design

Twenty-nine patients with bipolar disorder according to DSM-IV were recruited into the study, 16 females (mean age 33 years, range 19–56) and 13 males (mean age 36 years, range 22–58). Fifteen patients had the diagnosis of bipolar type 1, and fourteen patients bipolar type 2. All patients also met criteria for Major Depressive Episode.

Patients were started on lamotrigine as a part of their clinical regime. The design involved two visits: before lamotrigine treatment started and after 10–12 weeks, when clinical response was assessed. An MRS scan was performed at each visit. The lamotrigine was prescribed by a clinician responsible for management of the patient according to clinical guidelines and the patient's response to treatment.

Of the 24 patients who were scanned at baseline, in nine lamotrigine was their only drug therapy; in the other fifteen patients lamotrigine was added to other drug treatments, including citalopram, sertraline, fluoxetine, duloxetine, venlafaxine, mirtazapine, aripiprazole, quetiapine, lithium, and sodium valproate. This drug treatment continued unchanged for the duration of the study.

Clinical response to lamotrigine treatment was assessed by the Hamilton Rating Scale for Depression (HAM-D). HAM-D ratings were taken at baseline and at the second scan. Response to treatment was defined as a 50% decrease in HAMD score from baseline.

Baseline MRS data were available for twenty-four patients with response status known for twenty-two patients and MRS datasets for both the baseline and follow-up scan were available for twenty-one patients. Five patients were unable to complete the baseline MRS protocol due to high levels of anxiety in the scanner (four patients) and restless legs syndrome (one patient). Three patients did not return for the follow-up scan: one patient because of eye surgery, one where lamotrigine was stopped by their treating clinician, and one due to practical issues.

### 2.3 Proton magnetic resonance spectroscopy

**Fig. 1:**
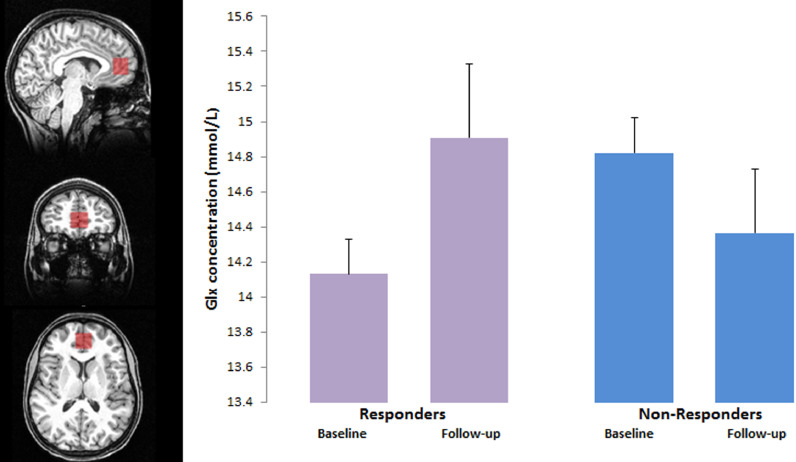
MRS voxel placement in the anterior cingulate cortex (ACC) and Mean (SD) Glx measures from this voxel in responders and non-responders to lamotrigine at baseline and after 10–12 weeks of treatment (‘Follow-up’).

Spectra were acquired using the Siemens Trio 3-Tesla whole-body MRI scanner and a 32-channel coil at the OCMR (University of Oxford). A high-resolution T1-weighted MP RAGE image was acquired for accurate MRS voxel placement and subsequent structural analyses (TR = 2040 ms, TE = 4.7 ms, flip angle = 8°, 192 transverse slices, 1-mm isotropic voxels). B0 shimming was achieved using a GRESHIM, available as a work-in-progress package on the Siemens system. Spectra were measured from the pregenual ACC voxel (2 × 2 × 2 cm^3^) (Fig. 1) with a semiadiabatic localization by adiabatic selective refocusing (SEMI-LASER) sequence (TE = 28 ms; TR = 3 s; 64 averages) with variable power radiofrequency pulses with optimized relaxation delays (VAPOR) water suppression and outer volume saturation (Tkac and Gruetter, 2005). Unsuppressed water spectra acquired from the same voxel were used to remove residual eddy current effects and to reconstruct the phased array spectra (Natt et al., 2005).

All MRS spectra were visually inspected for quality and between scan differences before inclusion. Mean full width at half maximum (FWHM) at the baseline scan was 0.080 (SD = 0.009), and at the follow-up scan 0.077 (SD = 0.016). Signal to noise ratio (SNR) at the baseline scan was 30 (SD = 0.6.4), and at the follow-up scan 31 (SD = 7.4). Cramér Rao Lower Bounds (CRLB) was 4.6 for both scans (SD, respectively, 1.7 and 1.1).

Metabolites were quantified with LCModel (Provencher, 1993) using the unsuppressed water signal as reference. Model spectra of metabolites were generated based on previously reported chemical shifts and coupling constants by the GAMMA/PyGAMMA simulation library of VeSPA (Versatile Simulation, Pulses and Analysis) (Tkác, 2008, Soher et al., 2011). Metabolites quantified with Cramér-Rao lower bounds (CRLB, estimated error of the metabolite quantification) >20% were classified as not detected.

T1-weighted structural images were brain extracted and tissue-type segmented using the Brain Extraction Tool of FMRIB Software Library (FSL) and Statistical Parametric Mapping (SPM) (Smith, 2002). The percentage of cerebrospinal fluid (CSF) within the MRS voxel was calculated from the resulting images, and used to correct metabolite concentrations for CFS fraction.

### 2.4 Statistics

Statistical analysis was performed using SPSS version 23. The effect of lamotrigine treatment on glutamate (Glx) levels in responders and non-responders was assessed with a two way repeated measures analysis of variance (ANOVA). Other comparisons were carried out with paired and unpaired *t*-tests and the Chi square test. Correlations were performed using Pearson's test.

## 3 Results

After 10–12 weeks’ treatment with lamotrigine, fifteen patients (68%) were classified as responders and seven (32%) as non-responders on the HAM-D. At this point, three patients were taking 100 mg, 150 mg, and 300 mg lamotrigine daily, as prescribed by their clinicians; all other patients were taking 200 mg lamotrigine daily. There were no significant differences between responders and non-responders with respect to age, gender, age at bipolar disorder onset and length of illness (Table 1).

**Table 1:** Demographic measures for patients for whom both valid baseline and follow-up MRS data were available. F, females; M, males; HAM-D, Hamilton Rating Scale for Depression.

		Responders *N* = 10	Non-responders *N* = 6	Statistics
Age (years)		36.9 ± 12.4	29.8 ± 11.3	*p* = 0.27, *t* = −1.14
Gender		F: 8, M: 2	F: 3, M:34	*p* = 0.21, *χ*^2^^ ^= 1.57
Age at onset (years)		21.2 ± 9.9	15.5 ± 5.3	*p* = 0.21, *t* = −1.29
Length of illness (years)		13.9 ± 6.1	14.3 ± 10.0	*p* = 0.73, *t* = −0.34
HAM-D score at baseline		21.0 ± 8.1	18.5 ± 4.5	*p* = 0.49, *t* = −0.71
HAM-D score at follow-up		5.5 ± 3.9	13.8 ± 4.8	*p* = 0.001, *t* = 4.14
HAM-D change during treatment	%	75.5 ± 17.5	26.8 ± 13.4	*p* = 0.000, *t* = −5.83

The mean ± SD HAM-D score at baseline was 20.5 ± 6.6 and did not differ between responders (20.8 ± 8.3) and non-responders (19.3 ± 4.6) to lamotrigine. At the time of the second scan the mean HAM-D score had fallen significantly (8.3 ± 5.9, t = 7.22, *p* = 0.001) (Table 1).

Sixteen patients had valid MRS scans on both scanning occasions. The ANOVA showed no main effect of lamotrigine treatment on Glx levels (*F* = 0.49, df = 1,14, *p* = 0.49); however, there was a significant interaction between response status and lamotrigine treatment (*F* = 7.51, df = 1,14, *p* = 0.016). Patients who responded to lamotrigine (*n* = 10) showed a significant increase in mean ± SD Glx levels over treatment (14.0 ± 0.90 vs 14.9 ± 0.99 µmol/g, *t* = 3.12, *p* = 0.012) while non-responders (*n* = 6) showed no significant change (14.8 ± 1.3 vs 14.3 ± 0.98 µmol/g, *t* = 1.11, *p* = 0.31). Overall there was no significant change in Glx level in ACC in the whole patient group (14.3 ± 1.1 vs 14.7 ± 0.99 µmol/g, *t* = 1.20, *p* = 0.25).

There were no differences in baseline Glx level between patients who went on to respond to treatment and those who did not, though responders showed a trend to have lower values (14.8 ± 1.2 vs 14.1 ± 0.93 µmol/g, *t* = 1.38, *p* = 0.19). There was no significant correlation between baseline Glx and change in HAM-D score over treatment (*r* = −0.31, *p* = 0.21).

## 4 Discussion

The main finding of this study is that lamotrigine, given over a period of about 12 weeks, significantly increased Glx in the ACC of depressed bipolar patients who responded to treatment, as defined by a 50% decrease in HAM-D core. However, there was no significant change in Glx levels in patients who did not respond. Also, lamotrigine did not change Glx levels in the patient group overall when baseline and follow-up scans were compared. These findings are clearly at variance with our hypotheses as stated in the Introduction.

Studies in animals suggest that lamotrigine decreases glutamate release, probably as a consequence of sodium channel blockade (Ketter et al., 2003). However, two previous MRS studies of lamotrigine in bipolar patients have not confirmed these animal experimental data. For example, Croarkin et al. (2015) observed a significant increase in Glx in the ACC of 15 bipolar patients after 12 weeks of lamotrigine treatment, though change in Glx did not correlate with therapeutic response. In contrast, Frye et al. (2007) found no change in ACC Glx levels in 23 depressed bipolar patients following 12 weeks lamotrigine. Again, there was no correlation between change in Glx levels and clinical response. Both the latter investigations were carried out at relatively low MR field strength (1.5 Tesla) which makes it difficult to interpret individual components of the Glx signal.

The notion that lamotrigine might lower ACC Glx in bipolar depressed patients is consistent with the view that bipolar depressed patients have raised Glx levels prior to treatment. While findings from different studies are not entirely consistent, meta-analyses have suggested that bipolar disorder is associated with increased brain Glx levels and specifically that bipolar depression might be distinguishable from unipolar depression on this basis (Gigante et al., 2012, Taylor, 2014). However, the latter possibility was not confirmed in a recent direct comparison between unipolar and bipolar depressed patients where both patient groups had lower glutamate levels than healthy controls (Wise et al., 2018). Because our study lacked a healthy comparison group we cannot comment on whether Glx levels in our patients prior to lamotrigine treatment differed from healthy controls. However, the fact that clinical improvement correlated with increased Glx might suggest that in these patients increased Glx was a consequence of the amelioration of depression rather than a direct result of lamotrigine treatment.

In this study we focused on the ACC, a brain region frequently used as a voxel in MRS studies of patients with mood disorders (Yuksel and Ongur, 2010) and in other MRS studies of lamotrigine treatment (see above). While the ACC has been strongly implicated in the pathogenesis of mood disorder (Cerullo et al., 2009) other relevant regions, such as thalamus and striatum, would also be worth investigating, as glutamatergic changes may be limited to individual structures. Hence it is possible that lamotrigine could alter Glx levels in brain regions other than ACC.

## 5 Strengths and limitations

The strength of the study is its application of MRS to measure the effect of lamotrigine on Glx in real world patients in a within-subject design. The limitations are the modest sample size, the fact that patients were also maintained on other medications, the lack of placebo control and that the MRS procedure was unable to separate glutamate and glutamine. One methodological issue for our study was that – as according to clinical practice - in most cases lamotrigine was added to pre-existing medication. Thus effects of these latter medicines on glutamatergic mechanisms might have modified or suppressed effects of lamotrigine. In particular other mood stabilisers such as lithium have been reported to influence glutamate neurotransmission (Sanacora et al., 2008, Yuksel and Ongur, 2010) perhaps making it difficult for a specific effect of lamotrigine to be detected.

Future studies would gain from the use of ultrahigh field MR (for example 7 Tesla) which yields clear delineation of glutamate and glutamine and thereby allows a better assessment of glutamatergic activity than is provided by the composite measure of Glx (Godlewska et al., 2017, Godlewska et al., 2018). Such studies should also include a larger initial number of patients, to increase the reliability of findings and to allow better for treatment drop-outs and technical failures with MRS imaging. Also a placebo control group would enable effects of lamotrigine to be more clearly separated from those relating to clinical change.

## 6 Conclusions

Our study shows no major effect of several weeks of lamotrigine treatment on Glx levels in the ACC in bipolar depressed patients. However, therapeutic improvement during lamotrigine was associated with increased Glx, suggesting that alteration in glutamatergic activity might be related to recovery from bipolar depression.

## Contributors

Beata R. Godlewska substantially contributed to conception and design of the study, acquisition of the data, data analysis and interpretation; she has drafted the manuscript. Uzay Emir substantially contributed in the area of methodological aspects regarding MRS data acquisition and analysis; he participated in drafting the manuscript. Charles Masaki contributed to data acquisition and analysis. Theodoras Bargiotas had substantial contribution to data acquisition. Philip J Cowen substantially contributed to conception and design of the study, data analysis and interpretation, and writing of the manuscript. All authors gave their final approval of the version to be published.

## Role of funding source

This work was supported by Oxfordshire Health Services Research Committee (grant number 1104) and Medical Research Council (grant number MR/K022202). CM was a Rhodes Scholar. The authors also acknowledge support from the NIHR Oxford Cognitive Health Clinical Research Facility. The views expressed are those of the authors and not necessarily those of the NHS, the NIHR or the Department of Health.

## Appendix Supplementary materials

Image, application 1
